# International perspectives on suboptimal patient‐reported outcome trial design and reporting in cancer clinical trials: A qualitative study

**DOI:** 10.1002/cam4.4111

**Published:** 2021-07-05

**Authors:** Ameeta Retzer, Melanie Calvert, Khaled Ahmed, Thomas Keeley, Jo Armes, Julia M. Brown, Lynn Calman, Anna Gavin, Adam W. Glaser, Diana M. Greenfield, Anne Lanceley, Rachel M. Taylor, Galina Velikova, Michael Brundage, Fabio Efficace, Rebecca Mercieca‐Bebber, Madeleine T. King, Derek Kyte

**Affiliations:** ^1^ Centre for Patient‐Reported Outcomes Research Institute of Applied Health Research University of Birmingham Birmingham UK; ^2^ NIHR ARC West Midlands London UK; ^3^ Birmingham Health Partners Centre for Regulatory Science and Innovation University of Birmingham Birmingham UK; ^4^ NIHR Birmingham Biomedical Research Centre and NIHR Surgical Reconstruction and Microbiology Research Centre University Hospitals Birmingham NHS Foundation Trust and University of Birmingham Birmingham UK; ^5^ GlaxoSmithKline (formerly of CPROR, University of Birmingham Birmingham UK; ^6^ UK National Cancer Research Institute (NCRI) Psychosocial Oncology and Survivorship CSG Subgroup: Understanding and Measuring the Consequences of Cancer and its Treatment London UK; ^7^ School of Health Sciences University of Surrey Guildford UK; ^8^ NIHR ARC Kent London UK; ^9^ Clinical Trials Research Unit University of Leeds Leeds UK; ^10^ Macmillan Survivorship Research Group Health Sciences University of Southampton Southampton UK; ^11^ N. Ireland Cancer Registry Centre for Public Health Queens University Belfast UK; ^12^ Leeds Institute of Medical Research at St James’s University of Leeds Leeds UK; ^13^ Sheffield Teaching Hospital NHS Foundation Trust and University of Sheffield Sheffield UK; ^14^ UCL Elizabeth Garrett Anderson Institute for Women’s Health Medical School Building University College London London UK; ^15^ Centre for Nurse, Midwife and Allied Health Profession Led Research (CNMAR University College London Hospitals NHS Foundation Trust London UK; ^16^ Queen’s Department of Oncology Queen’s Cancer Research Institute Kingston ON Canada; ^17^ Italian Group for Adult Hematologic Diseases (GIMEMA Health Outcomes Research Unit Rome Italy; ^18^ School of Psychology University of Sydney Sydney NSW Australia; ^19^ University of Sydney NHMRC Clinical Trials Centre Sydney NSW Australia; ^20^ School of Applied Health & Community University of Worcester Worcester UK

**Keywords:** cancer, patient‐reported outcomes, protocol, qualitative, reporting, trials

## Abstract

**Purpose:**

Evidence suggests that the patient‐reported outcome (PRO) content of cancer trial protocols is frequently inadequate and non‐reporting of PRO findings is widespread. This qualitative study examined the factors influencing suboptimal PRO protocol content, implementation, and reporting, and use of PRO data during clinical interactions.

**Methods:**

Semi‐structured interviews were conducted with four stakeholder groups: (1) trialists and chief investigators; (2) people with lived experience of cancer; (3) international experts in PRO cancer trial design; (4) journal editors, funding panelists, and regulatory agencies. Data were analyzed using directed thematic analysis with an iterative coding frame.

**Results:**

Forty‐four interviews were undertaken. Several factors were identified that could influenced effective integration of PROs into trials and subsequent findings. Participants described (1) late inclusion of PROs in trial design; (2) PROs being considered a lower priority outcome compared to survival; (3) trialists’ reluctance to collect or report PROs due to participant burden, missing data, and perceived reticence of journals to publish; (4) lack of staff training. Strategies to address these included training research personnel and improved communication with site staff and patients regarding the value of PROs. Examples of good practice were identified.

**Conclusion:**

Misconceptions relating to PRO methodology and its use may undermine their planning, collection, and reporting. There is a role for funding, regulatory, methodological, and journalistic institutions to address perceptions around the value of PROs, their position within the trial outcomes hierarchy, that PRO training and guidance is available, signposted, and readily accessible, with accompanying measures to ensure compliance with international best practice guidelines.

## Graphical abstract

Patient‐reported outcomes (PROs) capture patients’ experience of the physical, functional, and psychological effects of disease and treatment. However, research to date indicates the design and reporting of PRO data in cancer clinical trials are suboptimal. Our findings highlight a number of potential factors contributing to observed suboptimal PRO protocol quality and reporting in cancer clinical trials. We provide potential solutions to uphold best practice to improve the quality of PRO data for the benefit of cancer patients.

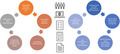

## 1 INTRODUCTION

Patient‐reported outcomes (PROs) enable the assessment of cancer, its impact, and treatment, from the patient's perspective, and are collected using validated self‐reported questionnaires. PROs provide valuable information, including data on physical symptoms, treatment toxicities, psychosocial problems, global health‐related quality of life, to assess treatment effectiveness, and tolerability.^1^, ^2^

Patient‐reported outcome data support informed decision‐making by patients from diagnosis and throughout treatment.^2^ Its value has been recognized by key stakeholders including clinicians, funders, regulators, and policy‐makers.^3^, ^4^, ^5^ However, the growing literature suggests that the quality of PRO data may be compromised^6^ through the omission of PRO‐related content in trial protocols.^7^, ^8^ Moreover, PRO data are often poorly reported^9^, ^10^, ^11^ or not included in trial publications.^12^ Despite this evidence, a substantial, and growing, number of randomized controlled trials (RCTs) across all cancer types now include PROs as primary and/or secondary endpoints.^13^, ^14^ Poor PRO protocol content and subsequent poor outcome reporting reduce the extent to which PRO results reach and inform clinical interactions and decision making, while simultaneously devaluing the contribution of trial participants providing this information. Qualitative methods have provided important insights into the challenges when administering PROs in trials.^6^ To date, there is limited qualitative research exploring the factors affecting PRO trial design, data collection and reporting, or patients’ and clinicians’ access to PRO data to inform decision making.

The Evaluation of Patient‐Reported Outcome Protocol Content and Reporting in UK Cancer Clinical Trials (EPiC) study used mixed methods to investigate PRO protocol content and reporting in a cohort of international cancer clinical trials. Phase I highlighted inadequate PRO protocol components, widespread non‐reporting of PRO trial results, and considerable delay and poor standards of reporting where PRO data were published.^8^ More than one‐third of the trials, involving 49,568 participants, failed to publish their PRO findings.

The current paper reports Phase II of the project: a qualitative study involving key stakeholders. Our aim was to examine and describe perceptions of the factors influencing suboptimal PRO protocol content, implementation, and reporting, including barriers, solutions, and examples of good practice.

## 2 METHODS

The study was completed according to the published protocol,^15^ conducting semi‐structured face‐to‐face, and telephone interviews between May 2017 and January 2018. The study was approved by the University of Birmingham Ethics Committee (Ref: ERN_17‐0085).

### 2.1 Recruitment

Purposive sampling was used to recruit participants from four stakeholder groups: (1) trialists and chief investigators with experience of cancer trials collecting a PRO as a primary or secondary outcome; (2) individuals with lived experience of cancer; (3) international experts in PRO cancer trial design, including members of international cancer and quality of life research organizations, national advisory bodies and industry; and (4) journal editors, funding panelists, and regulatory agency representatives (Appendix S1). Approaches were via email, either directly, where contact details were available in the public sphere, or via the center from which they were identified where appropriate. Recruitment continued on a rolling basis until data saturation was reached. Eligible individuals were provided with a brief outline of the study and those interested in participating were invited to take part in an interview. Participants completed and returned a consent form prior to the interview or gave verbal recorded consent.

### 2.2 Data collection

All interviews were conducted by a qualitative researcher (AR). The interviews were digitally audio‐recorded and professionally transcribed verbatim. A preliminary topic guide was formulated in advance of the interviews informed by the research aims, which were iteratively refined to explore emerging themes. Additional prompts were developed for each participant group as required (Appendix S2).

### 2.3 Analysis

Transcripts were analyzed by AR using directed analysis.^16^ Findings from previous qualitative and review work, coupled with insights into Phase I of the project, were used to develop an initial coding framework. A flexible and iterative approach was used to continually develop and refine the coding frame, allowing for the emergence of novel themes. Additional codes were developed and included as the analysis progressed and the framework modified accordingly.^17^ Transcripts were coded line by line and as the coding frame evolved, transcripts were recoded to ensure new themes were captured. The coding frame and sample codes were checked by DK and MC. Disagreements were resolved through discussion.^18^ NVivo 11^19^ was used for data management and to facilitate analysis.

## 3 RESULTS

The interviews lasted for 30–60 minutes. For recruitment, participants were assigned to one of the four pre‐defined stakeholder groups (trialists and chief investigators; those with lived experience of cancer; international experts in PRO design; and journal editors, funding panelists, regulatory agency representatives). However, several participants identified with or had features and experiences that resonated with more than one stakeholder group (Table 1).

**TABLE 1:** Qualitative study participant characteristics

Interview group (total) (n)		Country of employment (n)		Additional areas of expertize/experience (n)	
Trialists and Chief Investigators	10	UK	10	Research Clinical Patient & Public Involvement PRO expertise Methodologist (not PRO) Pharmaceutical experience	10 5 1 2 5 1
Lived experience of cancer	12	UK Spain	11 1	Regulatory Clinical Funding panelist Patient and Public Involvement	3 1 5 12
International experts in PRO cancer trial design	10	USA Belgium UK Netherlands	7 1 1 1	Regulatory Research Clinical Funding panelist Patient & Public Involvement Journal editor PRO expertise Methodologist (not PRO) Pharmaceutical experience	2 10 3 4 1 6 10 6 4
Journal editors, funding panelists, regulatory agency representatives	12	UK Canada USA Austria	8 2 1 1	Regulatory Research Ethics Research Clinical Funding panelist Patient & Public Involvement Journal editor PRO expertise Methodologist (not PRO)	3 2 7 7 5 4 4 1 5

Abbreviations: PRO, patient‐reported outcome; UK, United Kingdom; USA, United States of America.

Interviewees highlighted several barriers and facilitators to optimal PRO practice relating to study inception, PRO collection, analyses, data publication, and training and guidance (Table 2).

**TABLE 2:** Study phases and themes

Study phase and themes		Example quote and source identifier and primary stakeholder group
**1. Study inception**		
Barriers	Perceived pressure to include PRO	Generally, people tend to pick reasonable measures, reasonable, in cancer trials they do anyway, not always, I guess, but I think they are shoehorned in because everyone thinks they should fit in HTA type trials, they should be measuring the quality of life [013, Trialist/Chief Investigator]
	PRO included at a late stage	I have been contacted by lots of oncologists who say, “We're doing this trial but we thought we ought to tack on this questionnaire or that questionnaire. What do you think?” But it is been an afterthought and the rest of the protocol is really quite clearly established but they just want to add something a little bit extra but it is not the primary focus. I think that's a problem because until patient reported outcomes does become a primary focus for oncology research, then it won't get the attention it needs [020, Trialist/Chief Investigator]
	Skepticism about PRO	Maybe there is some reluctance by some to include them in that they could be considered far too subjective, I mean one particular study, we got involved in, there was a lot of debate about the use of some patient‐reported outcomes and that some critics were saying they are far too subjective and that having objective measures of mobility, would be far more useful [015, Trialist/Chief Investigator]
	Focus on survival	Oncology trials center on reduction in patients who are sick, it is about the reduction in survival. Patients are not necessarily expected to get much better. It is about reducing the time for progression or death. It is because, what is distinctive about oncology, especially in later or advanced cancer, it is about longevity and that is what ranks … It is not about well‐being, functioning, symptomatology, as much as it is about, of course, safety and tolerability are very important, that is first and foremost. But when it comes to the efficacy side, it is about survival, because of the nature of the disease … what is said to be the most meaningful outcome is those time to event outcomes. That is what the experts and other stakeholders say. [029, International Expert]
	Position of PRO in outcome hierarchy	They are usually always sub‐protocols; they are not built into the main trial design itself. Because the trials are perceived to be so burdensome anyway, that because if you think back to the importance in people's minds, it's kind of “the extra little bit,” it is the bit that usually gets cut first [043, Trialist/Chief Investigator]
	Data required for drug approval	In Oncology it typically has not been the case that a drug is approved for Patient‐Reported Outcomes. Its progression‐free survival, overall survival, event‐free survival, some type of “time to event” that the weight of the evidence falls on. If the Patient‐Reported Outcomes are reported typically, it serves to supplement and complement. It is not the main outcome of interest. [029, International Expert]
Facilitators	Formulating clear PRO rationale	One of the real key components is a company understanding early in the development program why they are going to collect the data and if they are going to collect it, and this really impacts the overall data quality that you see later on. If you have not decided earlier, the company or drug developer, that you are going to collect PRO and then you only decide later on in the development program when you realize that maybe a payer or someone else might ask for that data, usually the quality of the data, the hypothesis‐generating information and methodology is done quite poorly and it is quite difficult to conclude from that data [006, Trialist/Chief Investigator]
	PRO expertise in protocol development	[The PRO advisory group] make recommendations and will sometimes provide some sample packs … We do try to get as much of that sort of stuff into the protocol as we can, so that it is written in stone as … part of the protocol and any deviation from that is, a deviation to the protocol … administration processes, that are too detailed for the protocol might go into a site training document or an appendix that is used as instructional material or a checklist for the site [010, International Expert]
	Inclusion of patient perspective	Often the issues that researchers think are important to get out there, are not important to the patients. They are more interested in … quality of life measures that sometimes the researchers do not put such great store on. They are more bothered in whatever the primary end point was and it is very rare for a primary end point to be a quality of life. It is usually progression‐free survival or overall survival [033, Lived Experience]
	New focus on gentler treatments	The treatment has advanced to a point where is not just a matter of, can we keep you alive, but, can we keep you alive and comfortable, can we improve your survival and improve your symptoms, then it starts becoming more important to actually be able to assess the symptoms as opposed to when we have drugs that just, universally made people feel God awful. [010, International Expert]
	Quality of survival	I’ve had male patients who now say they wish they hadn't had radical treatments for prostate cancer, because of the, you know, what it's left them with, incontinence, impotence. Breast cancer patients, years on who have got lymphedema and can't lift their arms up and had debilitating things like that or weight gain from steroids. [033, Lived Experience]
	Identifying specific PRO aims	We listed, effectively, the areas that we wanted to cover. That was urinary symptoms, sexual function symptoms, bowel symptoms, and then generic aspects of quality of life, including anxiety and depression. Once we listed those key items, we then looked for questionnaires [028, Trialist/Chief Investigator]
**2. PRO collection**		
Barriers	Selection of PRO instruments	The metrics may not be as well established as say, as in cardiac output like that in the randomized trial so the metrics may not be as well established, there may be less agreement about how to measure things there might be more subjectivity and that can be harder to operationalize, even administration may be somewhat looser or less rigorous compared to some more biological outcomes that can be standardized and where timing can be adhered to more so I think just describing the outcomes might be a bit more challenging, the fact that the outcomes may be less well‐established, it can be a challenge sometimes [004, Journal Editor/Funder/Regulator]
	Balancing participant burden and trial aims	There would also be something around the burden. A lot of [ethics committee members] look at the burden on the participant, if they are being asked to fill in or complete too many questionnaires [041, Journal Editor/Funder/Regulator]
	Site staff capacity	We are always conscious that nurses and doctors never have enough time to do anything at all. So actually many of us feel guilty about the amount of time we are spending talking with doctors and nurses, even if it is answering the questions on a survey. I have done this. I have been sitting on the ward filling out my survey and someone on the bed opposite started to throw up and I just thought, “My nurse should really be going and attending to them. Their needs are more important. Come back to me.” And actually, I cannot even remember whether we finished the survey or not but that is the hard reality of frontline work [007, Lived Experience]
	Expense associated with PRO	The pain for this condition subsides in 4 days, so we proposed to develop a diary and they won't buy it. They are going with just this one [questionnaire, once a] month … Because they do not trust us to do the development. It is too costly. It is going to take up too much time and after all, it is just another end point [001, International Expert]
	Signaling of PRO importance by trialists	If it is not much in the protocol, people do not necessarily think it is that important, right? They pay attention to what they are reading and … the more word count … the more explanation and the more obvious it is considered important and people sometimes just do not see or see the value of what is hardly listed [008, International Expert]
Facilitators	Monitoring missing data	In the trial … we had to find, a priori, how we would deal with missing data. We also made a very substantial effort, at the time the trial was ongoing, not to accept the PRO back from patients until they had completed it and we chased patients quite hard when they did not complete it. So actually, the amount of missing data was very small [020, Trialist/Chief Investigator]
	Buy‐in from trial staff	If you have it in protocol and you are ready to do this, then you have to convince the people who are collecting the patients for you, that it is also important to have this data, to have these questionnaires filled out. That is another really problematic hurdle … because patients are willing to do this. But it is mainly the personnel and the daily business, that you are too busy to do all these things and to discuss this with the patients. You need nurses, you need facilitation for people to help you in collecting these data. And that is important. And also to keep up the compliance over time. And that is, I think the second hurdle because missing data is one of the main issues which is going to, well, to negatively affect the impact of the PRO measures in a clinical trial [023, International Expert]
	Engaging trial participants	There has to be some responsibility obviously on the patient, it has to be a shared task so improved tech partnership working is what is so important that the patient feels a part of the team and actually your data is incredibly valuable to us because it will inform research and clinical practice you know as an ongoing issue and it may be years down the line before you may see the benefit but you will be contributing to this so you are part of our team [009, Lived Experience]
	Use of innovative PRO delivery	We have the technology to do all sorts of interesting things in real‐time, the reporting and recording of data… So I have become more and more interested, PROs and actually what they could be rather than what they are [007, Lived Experience]
**3. Analyses**		
Barriers	Planning analyses	Which are the key items to look at? Because a major problem, when you use lots of different questionnaires that cover the same issue, is which one do you count on? … I think it is another problem with patient‐reported outcomes that you have these issues to deal with; the key items that everybody wants to know about or the key questionnaires and then there is a whole raft of other things that you have collected. It is not entirely clear how you deal with all that [028, Trialist/Chief Investigator]
	Team pressures	I think part of the problem, particularly with investigator‐sponsored studies where you have limited resources, even if you have infinite amounts of money, you still have limited resources and clearly, the focus is to get the most important data out there as quickly as possible because we want to make progress as quickly as possible. So, the Overall Survival data are the things you want to get clean, tidy, in, analyzed and presented first and so often, you end up saying, “Actually, we're going to focus on that and we're not going to bring in the Quality of Life data” or “We're not going to clean those up as a priority.” [018, Trialist/Chief Investigator]
	Missing data	In one of my studies, a randomized phase II/III we had substantial problems with missing second, third, and follow‐up reports and then we ended up going from the outset of 80% to below 40% in the follow‐up and it was nearly impossible to draw any conclusions. [040, Journal Editor/Funder/Regulator]
Facilitator	Handling missing data	I think it was discussed with the statisticians … it was probably stated in the protocol how we would [manage missing data]. Again, I think to have an a priori approach to what you are going to do may help minimize bias when you do report the data [020, Trialist/Chief Investigator]
**4. Data publication**		
Barriers	Results of other outcomes	Very often, when … the trial is not positive, often the Quality of Life data never sees the light of day. That is a shame, although I guess it is probably not going to be hugely important because if your primary end point is negative, you are probably not going to change practice. It is unlikely that you would change practice based on a secondary end point based on patient‐reported outcomes. I think it is often just expediency. [018, Trialist/Chief Investigator]
	Quality of PRO data	The … trial group decided not to publish it at all because you lose your reputation when you publish bad data so this is something that you usually should not do and also not try to publish it in a bad journal, so putting bad data into a bad journal I think is a bad idea. [040, Journal Editor/Funder/Regulator]
	Selective reporting	I think that it may be a rush to statistically significant results, investigators may feel that, “oh, this Patient‐Reported Outcome is not statistically significant. It may dampen our ability to seek additional funding. It may dampen the perceived effectiveness of the intervention.” [031, Journal Editor/Funder/Regulator]
	Ranking of PRO among outcomes	If the PRO were only an exploratory end point within the protocol sometimes they are not even mentioned so they might have been mentioned as, “These were end points,” but then you do not actually find any of the results or data actually in the publication at all. [006, Trialist/Chief Investigator]
	Perceived interest in PRO findings	If you look at any of the main clinical trial report articles there might be a small paragraph on the quality of life outcomes regardless of whether they are really good or just no difference between the treatments. Mainly because the interest in both is on survival, progression‐free survival, response rate, things of that nature which, and toxicity which seems to take more precedence. [005, International Expert]
	Perceived disinterest from high‐impact journals	If survival's not there … the “harder outcomes” are not there in your paper, you do struggle. We start when we try to send papers for publications as high an impact factor as we can go but we usually know that we are going to get rejected. We get rejected by Lancet Oncology, for example. It is a classic. You just get a little pat on the head but, “Oh, send this to a nursing journal.” British Journal of Cancer, some of the big names in the cancer field do not want to publish this sort of work so we work our way down the list. [021, Trialist/Chief Investigator]
	PRO data in secondary papers	As a secondary publication from a trial, unless it showed something really novel or really dramatic, it is unlikely and you do not really see Quality of Life data, patient‐reported outcomes, secondary measures as secondary publications being published in high‐impact journals. [018, Trialist/Chief Investigator]
	Journal constraints	The biggest problem here is that in 95% of the Oncology trials, Quality of Life is a supporting end point; so it is a secondary end point. That is a big barrier because when you want to publish the article, often if it is a secondary end point and there is nothing in there, then it might just end up being one paragraph or two paragraphs with a table in the journal … And so, we have faced the challenge before where journals say, “We want primary end point as much as we can and then we want you to publish a secondary end point but you only have 2,500 words,” That is extremely difficult. [002, International Expert]
Facilitators	Presence of PRO publication plan	We have always had an analysis plan [for the PRO] and a dissemination plan for how we would proceed, how we would analyze the data, how we would publish the data, how we would divide the data up, and what sort of papers we would write. [021, Trialist/Chief Investigator]
	Accountability to stakeholders	The other thing … is about encouraging patient and public involvement in the clinical trials. You do that from the very beginning, in terms of developing what the patient‐reported outcome measures are … then those people should be their conscience and should be saying, “Where are the results of this particular outcome measure that we worked with you on?” It becomes much more difficult, I think, for them to hide things if they have got a working group of people, or Working Advisors on the Steering Group, and to start backing out and not reporting things. [041, Trialist/Chief Investigator]
	Supplementary information	If you are collecting data in PRO or you are collecting data in any point, end point, you know, there should always be space either in the article or as supplementary information, you know, if there was not space to actually include the data or at least a summary of the data in the publication. [006, Trialist/Chief Investigator]
	Publication based on merit	If you write a good paper and you pitch it at the right journal, there is no reason why it should not get published, but it depends on how you write it and you have to put it in the right, you have to pitch it appropriately and you have to reflect the journal. [013, Trialist/Chief Investigator]
	Publishing in the main paper	It is detrimental to publish it separately because often it gets published separately, … months later; we have done a study where we looked at the difference in publication times and it was like, on average, 18 months later or something, and it is on average, in a [low] impact factor journal, much less than the primary outcomes and so I think the chances there are that the Clinicians reading it and using that data are not high, so even though people want to publish lots of papers, I think it would be better to say to them, publish the PRO results in the main trial paper and force them to write them succinctly. [013, Trialist/Chief Investigator]
	Demand for PRO data	Well there is actually a big hype around the quality of life data and usually, it is pretty easy to publish good quality PRO’s in the journals and there are some journals who really like to have the quality of life data of large‐scale randomized trials, that are the secondary end points, for example, usually, this is highly cited, and so it becomes pretty easy but it depends on the trial and the area, there are other areas where there is a lot of clinical interest and then it becomes easy to publish. If it is an area where no one is actually interested in and the clinical trial does not actually tell us anything then the quality of life data is not interesting either. [040, Journal Editor/Funder/Regulator]
**5. Training and guidance**		
Barriers	Supply of PRO expertise	I still think there is possibly a lack of expert knowledge around the [UK]…just not enough people; we have just had a q‐hour meeting with this Neurosurgeon who was embarrassed that he was asking me about PRO’s and he has not got a clue…There is still a lack of basic knowledge and expert experience, and I still get asked by trial teams, will you please be the PRO expert on our trials, you know, I have not got time, but I think, cannot you just do it yourself, it is not that difficult, but they clearly do not really know how to do it. [013, Trialist/Chief Investigator]
	Burden upon researchers	I think, as a researcher and especially as a single‐handed researcher, that the bureaucracy and the paperwork are overwhelming and have become increasingly so over the last ten years. It might have modest benefits in some areas but it certainly can hold people back. I think if there is guidance, especially if it is mandatory guidance and it is impossible to publish if you do not follow that guidance, then you have to really support researchers to access that sort of stuff, rather than putting burdens in their way. [020, Trialist/Chief Investigator]
	Need for specialized training	I do not know if there is a specific training, I think it is just … being very clear, what are the aims of your study, are you collecting the outcomes that you need to meet those aims, so I do not know whether there is specific training required other than, being trained to put together a good research proposal that is going to get funded. [015, Trialist/Chief Investigator]
Facilitators	Availability of PRO information resources	There is a lot of uncertainty about particularly which primary outcome to pick and which PROs are needed but are not going to be overkill and mean that the patients start dropping out of the study because they do not want to go through a massive questionnaire booklet. So if the training had the right content and … was delivered in a flexible way so that people could easily access it, whether that is webinars or what, I do not know, but yes, I could see a role for it. [016, Trialist/Chief Investigator]
	Integration of guidance	I guess by journals insisting on it being done in that way. Most journals insist on the CONSORTs. They could also mention CONSORT‐PROs then that would a help … I should really know more about the fact that the CONSORT has a PRO. I really did not know that. I had heard about the other ones. [017. Trialist/Chief Investigator]
	Upholding best practices	I think it is a multi‐stakeholder responsibility … physicians should demand it, clinicians should demand it, regulations should demand it, industry themselves should demand it, the payer should demand it. I think all of us have a responsibility to ensure that the information that we collect during a drug development program is sufficient but also the best it possibly can be to determine what the benefits and the risks are [006, Trialist/Chief Investigator]

### 3.1 Study inception

During study inception, interviewees reported that PROs were often included in discussions at a late stage; or added as an “afterthought” to meet funding requirements for example, and were considered to be of lower importance in the hierarchy of trial outcomes. The relevance and priority of PRO endpoints in trial design were considered dependent upon the clinical characteristics of the cancer type and the nature of the intervention. Participants described how, where interventions were intended to prolong life, survival would be a key outcome, and PRO data may be of lesser importance to the research question. In cases where a range of curative interventions was available or in a palliative setting, PRO data had greater prominence. Other respondents commented on difficulties associated with selecting appropriate PROs in trials and perceived reluctance by some trial investigators to include PROs owing to concerns regarding their subjectivity or cost. Recommendations to address these issues included: the need to formulate a clear PRO rationale early in the trial design process which incorporated the patient perspective; identifying specific PRO aims and objectives; and ensuring involvement of PRO expertise during protocol development. Participants described how advances in cancer treatment supported more curative options, development of less toxic treatments, and greater awareness of the effects of living with consequences of radical interventions, leading to a growing awareness of PRO data and its place in cancer trials and clinical practice.

### 3.2 PRO collection and analyses

Identified barriers centered around: perceived lack of standardized PRO administration compared to more “objective” clinical trial outcomes; concern around participant and staff burden associated with PRO completion; and a lack of communication with data collection staff regarding the importance of PRO data to the trial. Missing data were considered to be a significant challenge for analyses and concluding data. Subsequently, there was a view that the volume of missing data and its perceived poor quality could contribute to underreporting due to possible damage to reputation and limiting future funding prospects. Interviewees also highlighted a perceived lack of clarity surrounding PRO analysis methods as a key barrier. Others felt that a lack of trial resources often resulted in the prioritization of the primary outcome at the expense of PRO data.

Facilitators to optimal implementation of PRO within trials included: ensuring adequate PRO coverage in the protocol to help foster “buy‐in” from trial staff; engaging research participants by communicating the importance of their PRO data; development of a priori plans to minimize avoidable missing PRO data by identifying poor PRO compliance in real‐time, establishing statistical management of missing PRO data in advance, and pre‐specifying PRO analyses; and the use of innovative data‐capture technology to reduce burden and increase data quality.

### 3.3 Data publication

Discussion focused upon academic publication rather than inclusion in regulatory submissions or Health Technology Assessment applications. Interviewees highlighted issues around selective reporting of PROs, either linked to the significance of the primary outcome or of the PRO itself, or due to the “lower ranking” of PRO results by trialists. There was also a perception that PRO findings were of little interest to journals unless particularly “novel” or “dramatic,” and that the inclusion of PRO data alongside the primary outcome could be impeded by restrictive word count limits. Interviewees discussed solutions including the generation of a dissemination plan that includes PROs; encouraging Patient and Public Involvement (PPI) throughout the trial to foster accountability and promote complete reporting; and the availability of the option to include PRO data as supplementary files or appendices. When asked about perceived demand for PRO data from journals, participants who had successfully led and published research using PROs in high‐impact journals described how well‐written papers aimed at appropriate journals would get published. Participants, including those employed as journal editors, noted that journals are keen to publish high‐quality PRO data, particularly those from large‐scale RCTs, depending on the clinical area.

### 3.4 Training and guidance

A recurring theme was the perceived difficulty of acquiring and providing PRO training in practice. Respondents felt this was due to the lack of awareness of training resources; possible expense; limited time; and particularly for generalists, the notion that PROs were one of a multitude of competing for potential training needs, or that PROs were not within their remit. However, the majority of interviewees felt there was a role for all institutions involved in regulatory, funding, educational, methodological, and journalistic activities throughout the research process to ensure that PRO training and guidance was available, signposted, and readily accessible to stakeholders, with accompanying measures to ensure adherence and compliance to best practice guidelines.

### 3.5 Additional themes

There were two unanticipated themes resulting from the interviews (Appendices S3 and S4), drawn from all stakeholder groups. The first relates to the usefulness of PRO data in meeting the information needs of people during diagnosis, treatment, and when living with and beyond cancer (LWBC). When asked about their experience of accessing PRO information, several participants with lived experience described how the availability of PRO data during clinical decision making would have enabled more informed treatment choices. The second related to the generation of data valued by patients through PROs in cancer clinical trials, via effective PPI in study question formulation; development and use of PRO measures that are relevant and reflect patients’ interests; and the simplification of PRO delivery to minimize patient burden and improve engagement (Appendices S3 and S4).

## 4 DISCUSSION

Existing research has drawn valuable insights into the challenges faced when administering PROs in trials^6^; however, the current study is the first investigation of factors affecting PRO trial design, data collection and reporting, or patients’ and clinicians’ access to PRO data to inform decision making. Our findings highlight a number of potential factors contributing to observed suboptimal PRO protocol quality and reporting in cancer clinical trials: low prioritization of PROs; lack of training; late inclusion of PROs in trials; uncertainty related to analysis; and poor implementation (leading to missing data and interpretation issues). These span all phases of trial development, implementation, and dissemination and require the concerted and coordinated effort by the cancer research community to address them, for the benefit of all cancer patients. A particular focus should include addressing perceptions around the value of PROs and their position within the hierarchy of trial outcomes, and adoption of international guidance around best‐practice PRO trial design, data capture, analysis, and reporting.

Missing data were a recurring theme and was posited as a reason for skepticism around the value of PROs. This is reiterated in the literature, where high rates of missing PRO data continue to be reported.^10^ It is hoped that the widespread adoption of recent guidance around design and measurement selection, implementation, and reporting strategies aimed at reducing the instance and impact of missing PRO data will support more effective capture of PRO trial data in the future.^20^ Recent international cancer trials have successfully collected PRO data on a large scale, while demonstrating the ability to do so with very little missing data, minimal resource costs, and negligible investigator burden.^21^ PRO data are being successfully collected in challenging trial settings, for example, those performed across several centers and including highly vulnerable cancer populations.^22^ The need to include patients in the development, application, evaluation, and interpretation of PROMs is well established,^23^ to ensure their acceptability and relevance.^24^

A number of interviewees also supported the a priori development of statistical analysis plans including methods to address missing PRO data. The imminent publication of guidelines arising from the Setting International Standards in Analyzing Patient‐Reported Outcomes and Quality of Life Endpoints (SISAQOL) Data Consortium should help support trialists to implement this recommendation.^25^

There were diverging views around the non‐publication of PRO data.^8^, ^12^ While several researchers cited the experience of journals’ general reluctance to publish findings, particularly in primary trial manuscripts, interviews with journal editors suggested the route to publication was influenced instead solely by the quality of the individual manuscript. It is difficult to reconcile these opposing viewpoints. Open access international guidelines are available via the Consolidated Standards of Reporting Trials (CONSORT) PRO Extension,^26^ based on the methodological framework for guideline development proposed by the Enhancing the Quality and Transparency of Health Research (EQUATOR) Network.^27^ Intended to support researchers in raising standards of PRO reporting and if the broader pool of journal editors share the views of our interviewees, this could increase the probability of publication. Recent evidence also suggests an association between improved PRO reporting quality and the quality of the trial protocol,^8^ development of which may be aided by the Standard Protocol Items: Recommendations for Interventional Trials (SPIRIT),^28^ and the SPIRIT‐PRO Extension^29^ international consensus guidelines. Widespread endorsement and adoption of these guidelines by trialists, journals, funders, and regulators would help drive up future standards of PRO reporting through implementation initiatives such as the Patient‐Centered Outcomes Research Institute (PCORI) funded Patient‐Reported Outcomes Tools: Engaging Users and Stakeholders (PROTEUS) Consortium.^30^ Increased interest in PROs is echoed in the work of regulators, such as the Food and Drug Administration (FDA) recent patient‐focused drug development initiative,^31^ and the European Medicines Agency (EMA) guidance on the use of PROMs in cancer trials.^5^ Despite this, evidence suggests that the impact of PRO data is limited.^32^, ^33^ Issues relating to PRO‐related training are identified as a key theme, despite the availability of PRO training resources,^34^, ^35^, ^36^ demonstrating the need to enable and uphold greater uptake.

In summary, our interviewees suggested future cancer trials should include more comprehensive PRO trial design and protocol development involving PRO expertise and patient input, with a focus on standardized administration. They also emphasized the need to minimize the burden for patients and staff; prevent missing data; address missing data with appropriate analysis methods; develop a priori PRO analyses and dissemination plans; and train staff. The use of PPI throughout a trial life‐cycle was identified as integral to ensuring PRO data generated through cancer clinical trials are relevant and accessible to people with cancer. Unfortunately, the study findings suggest that stakeholders perceive and observe barriers to the interpretation and dissemination of PRO results, and that key factors that arise during the design and data collection of cancer clinical trials compromise this process.

### 4.1 Clinical implications

Our qualitative results suggest that the specific features of cancer and treatment affect whether PROs are placed lower in the trial outcome hierarchy as compared to survival outcomes. This appears to shape the expectations of those in the field, conceptualizing trials and determining research questions, perpetuating the relatively low position of PROs in the trial outcome hierarchy. However, the interview findings also suggest that the relative position of PROs in the cancer trial outcome hierarchy could be changing over time. Participants described the growing demand for PRO data due to increasing awareness around the impact of cancer on individual quality of life and the considerable burden associated with the acute, medium‐term, and late effects of treatment. Participants described witnessing relatives and friends undergoing treatment and the lasting long‐term consequences and how this informed the type of information they would seek while considering treatments for themselves. This is echoed in studies outlining the research priorities of those with lived experience of cancer,^37^ reiterating the need to integrate meaningful PPI when setting the research agenda. Early and consistent PPI may lead to a shift in emphasis from survival or cancer progression in isolation to also include the quality of survival; patient‐centered PRO rationale, aims, and objectives; selection of meaningful PROs, thereby making subsequent findings potentially more impactful; enhancing communication with participants around trial PROs; maximization of trial feasibility, recruitment, and retention; and minimization of PRO burden, participant study drop‐out, and missing data.^20^, ^38^, ^39^, ^40^

### 4.2 Study limitations

The strength of this work lies in the use of rigorous methodology; the broad expertise of the interviewees; and the inclusion of numerous stakeholder groups. However, a limitation is that international recruitment efforts were focused on the PRO and cancer methodology experts rather than across all stakeholder groups. A further limitation is that the study was at risk of self‐selection and social desirability bias, whereby participants participated due to a pre‐existing interest in PROs, portray their behaviors positively due to the nature of this study, with increased awareness of methodological issues and need for training. Despite this, the divergence of opinions identified indicates that individuals were sampled with a wide range of views related to PROs. Another possible limitation is that through the inclusion of people with lived experienced of cancer, there may be a bias toward the views of those who have survived and are LWBC. Their perspectives are more likely to reflect issues that pertain to the long‐term impacts of cancer treatments. Similar studies in the future may benefit from the inclusion of people affected by cancer more broadly, including carers, and those who are bereaved.

## 5 CONCLUSIONS

Our aim was to explore with stakeholders the factors influencing suboptimal PRO protocol content, implementation, and reporting. These qualitative findings suggest that a lack of training, understanding about the value of PRO data (that results in low prioritization of PROs in outcome hierarchy), difficulties associated with the numerous ways to analyze PRO data, and the expertise required to this end can undermine their planning, collection, and reporting can undermine their planning, collection, and reporting.

## CONFLICT OF INTEREST

DK, AR, KA, TK, and MC were supported by project funding from Macmillan Cancer Support. JA, LC, AGa, AGl, DK, AL, RT, and DG are all members of the National Cancer Research Institute Psychosocial Oncology and Survivorship CSG subgroup: Understanding and measuring the consequences of cancer and its treatment. AR is partially funded by NIHR Applied Research Collaborative West Midlands. MC is a National Institute for Health Research (NIHR) Senior Investigator and receives funding from the NIHR Birmingham Biomedical Research Centre, the NIHR Surgical Reconstruction and Microbiology Research Centre at the University Hospitals Birmingham NHS Foundation Trust, NIHR Applied Research Collaborative West Midlands, Health Data Research UK, Innovate UK (part of UK Research and Innovation), Macmillan Cancer Support, UCB Pharma, GSK, and the Patient‐Centered Outcomes Research Institute (PCORI). The views expressed are those of the authors and not necessarily those of the NHS, the NIHR, or the Department of Health. MC has received personal fees from Astellas, Takeda, Merck, Daiichi Sankyo, GSK, and Glaukos outside the submitted work. MC has led the development of SPIRIT‐PRO and CONSORT‐PRO international guidance and is a member of the SISAQOL and PROTEUS Consortia. LC has received personal fees from Boehringer Ingelheim outside the submitted work. JA receives funding from EU FP7, NIHR HS&DR, Chief Scientist's Office, Scotland, Macmillan Cancer Support. FE reports consultancy for Abbvie, Amgen, Janssen, Orsenix, Takeda, and grants from Amgen (to his Institution), outside the submitted work. MTK is supported by the Australian Government through Cancer Australia. MTK has received funding from the Australian National Health and Medical Research Council, ABBVIE, and BMS for research unrelated to the submitted work. MTK co‐led the development of SPIRIT‐PRO international guidance and is a member of the SISAQOL and PROTEUS Consortia. JMB receives funding from NIHR, YCR, and CRUK. DK reports grants from Innovate UK, the NIHR, NIHR Birmingham Biomedical Research Centre, and NIHR SRMRC at the University of Birmingham and University Hospitals Birmingham NHS Foundation Trust, and personal fees from Merck outside the submitted work. RMT receives funding from the NIHR Economic and Social Research Council (ESRC), Sarcoma UK, and UCLH Charity. DG and RMT are National Institute for Health Research (NIHR) Senior Nurse Research Leaders. GV is a University of Leeds Professor of Psychosocial and Medical Oncology and Consultant in Medical Oncology at St James's University Hospital, The Leeds Teaching Hospitals Trust and receives funding from Breast Cancer Now, NIHR, EORTC, Yorkshire Cancer Research, Pfizer, and IQVIA. GV reports personal fees from Roche, Eisai, Novartis, and Seattle Genetics outside the submitted work. GV is Chair of the NCRI Living with and Beyond Cancer group and is a member of the board of the EORTC. The views expressed in this article are those of the authors and not necessarily those of the University of Leeds, the NHS, the NIHR, or the Department of Health and Social Care.

## Supporting information

Appendix S1‐S4

## Data Availability

The data that support the findings of this study are available from the corresponding author upon reasonable request.
