# Supplementary material for: International perspectives on suboptimal patient‐reported outcome trial design and reporting in cancer clinical trials: A qualitative study
Source: Cancer Med. 2021 Jul 5;10(16):5475–87. doi: 10.1002/cam4.4111 (PMC8366078; doi:10.1002/cam4.4111)
Supplement: Supplementary file 1 — Appendix S1‐S4 [file CAM4-10-5475-s001.docx]

**Supplemental Appendix S1 - Purposive Recruitment Strategy**

**Stakeholder group targets:**

Aim: 12 participants per group

Identify 12 primary potential participants and 10 extra

**Demographic targets:**

Aim: approximately 20% Black and Minority Ethnic participants (BME); 50% gender split

Purposive sampling will be used on a rolling basis and individuals will be recruited based on the requirements of the sample to date.

**Group 1:**

**Format for sampling studies from Phase I**

Select 12 studies per domain (total 36):

1. **Clinical area** – 19 cancer types in total – randomly selected 12 from this list.
2. **Score** – 3 studies with highest PRO protocol checklist score; 3 studies with lowest PRO protocol checklist score; 3 studies with highest CONSORT PRO score; 3 studies with lowest CONSORT PRO score
3. **Funding source** – 3 studies with private funding; 3 studies with public funding; 3 studies with charity funding; 3 studies with mixed funding.

**Group 2:**

National Cancer Research Institute (NCRI) Consumer Liaison Groups and supplement with known PPI networks

ADDITIONAL AIM: Recruitment of BME participants via BME cancer research and support groups, and known contacts

**Group 3:**

International Cancer Research organisations/networks, International Quality of Life organisations/networks, leading pharmaceutical companies – cross-ref. with known contacts

ADDITIONAL AIM: Recruitment of perspectives of those with experience of collecting PRO data from BME communities; international settings; and regions besides North America and Europe

**Group 4:**

JOURNALS: Identify key journals for general medicine, cancer, and quality of life research – contact Editors and Deputy Editors

FUNDERS: Identify key funding bodies in UK for general research and cancer – cross ref. with known contacts

REGULATORS: Identify key regulatory agencies – cross ref. with known contacts

**Supplementary Appendix S2 - Topic Guides**

*Telephone interview topic guide*

**Introduction**

1. Re-state purpose of the interview: *Discuss your experience and understanding of Patient-Reported Outcomes in clinical cancer trials.*
2. Any questions or clarifications before beginning? If appropriate, have participants’ scoring information, protocol or publication for reference.

**Topics to be covered:**

**Please could you tell me a little about your background and your experience of PROs?**

[Consider which of the questions are of most relevance to participant and their expertise but cover all topics]

**What do you think are the factors that affect whether information related to PROs are included in trial protocols?**

*What are the barriers to including PRO information in protocols?*

*What enables the inclusion of PRO information in protocols?*

*How do you think inclusion of PRO information in protocols can be encouraged?*

**What do you think are the factors that affect whether PROs are effectively implemented during trials?**

*What are the barriers to implementing PROs during trials?*

*What enables the implementation of PROs during trials?*

*How do you think implementation of PROs during trials can be encouraged?*

**Have you any experience of collecting PRO data from minority groups in cancer clinical trials?**

*Any thoughts about encouraging their involvement?*

*Any barriers or enablers? Challenges or opportunities?*

*Translation of PROM into different languages/cultural adaptations?*

**What do you think are the factors that affect whether PRO information is appropriately reported in the resulting peer-reviewed publications following a trial?**

*What are the barriers for reporting PRO information in subsequent publications?*

*What enables the appropriately reporting of PRO information in subsequent publications?*

*How do you think the appropriately reporting of PRO information in subsequent publications can be encouraged?*

**Are you familiar with any guidelines or training that are specifically intended to assist when including PRO information in protocols?**

*If yes, please could you tell me more about them?*

*If no, do you think guidelines or training would be helpful? How could they be implemented?*

**Have you ever received any PRO-specific training?**

*If yes, please could you tell me more about the training you have received?*

*What was good about the training? What was bad?*

*Can you think of any examples of particularly effective/ineffective PRO-specific training you have received?*

*If no, do you think PRO-specific training would have been helpful?*

*What prevented you from receiving training?*

**Have you got any experience of providing training yourself?**

*If yes, please could you tell me about it?*

*What do you think are the key components of PRO training?*

**Are you familiar with any online PRO-training resources?**

*If yes, please could you tell me a little about them?*

*If no, do you think these would be helpful to your or your colleagues?*

**Are you familiar with CONSORT PRO reporting guidelines?**

*How do you think these guidelines can be made more accessible?*

*Do you think these guidelines could be better implemented? How?*

**Finally, would you say you are optimistic about the collection and reporting of PROs in cancer clinical trials in future?**

**OPTIONAL QUESTIONS**

1. **If scoring relevant scoring information is available:**

**[Referring to scoring information] Please could you explain how you decided which PRO related information to include in your protocol/publication?**

*Is there any further guidance you would have wanted to aid your decisions about what to include?*

*Are there any PRO related components of this protocol/publication that you would change, if given the opportunity?*

1. **For participants in senior research positions/journal editors**

**What do you think are the barriers/enablers for offering PRO-specific training to researchers who are working on trials/associate editors and peer reviewers?**

*Have you experienced any of these challenges in your own work?*

*In your experience, how could this kind of training be promoted?*

1. **For participants with experience of being a patient participant in research or improving patient engagement**

**What do you think can be done to encourage patients/participants to take part in the PRO component of a trial?**

*What are the challenges/motivators?*

*What would encourage more co-design?*

*Have you seen PRO results as a patient/used PRO data in treatment decisions? Where?*

**Are there any other aspects of PROs in clinical cancer trials that you would like to discuss?**

**Thank you for taking part.**

*Telephone interview topic guide- Phase I interviewees*

**Introduction**

1. Re-state purpose of the interview: *Discuss your experience and understanding of Patient-Reported Outcomes in clinical cancer trial and your recollections of the XXXXX trial*
2. Any questions or clarifications before beginning? If appropriate, have participants’ scoring information, protocol or publication for reference.

**Topics to be covered:**

**Please could you tell me a little about your background and your experience of PROs?**

[Consider which of the questions are of most relevance to participant and their expertise but cover all topics]

**Please could you tell me what you remember about how the PRO component of trial XXXXX was developed? [or general experience of planning PRO trial components?]**

*Was there someone specifically in charge of the PRO work?*

*Can you remember how the PRO measure was selected?*

*Can you remember how it was decided what would be included in the protocol?*

*Did you feel the team was equipped to do this or did you experience any challenges?*

*Is there anything about the PRO study design that you would do differently/do differently now?*

**Please could you tell me what you remember about how PROs were collected in the trial? [or general experience of PRO data collection?]**

*Do you remember any procedures for reducing missing data?*

*Do you remember any statistical methods for dealing with it?*

*Did you feel the team was equipped to do this or did you experience any challenges?*

*Is there anything about the PRO collection that you would do differently/do differently now?*

**Please could you tell me what you remember about collecting PRO data from minority groups in the trial? [or general experience of involving minority groups?]**

*Do you remember any processes to encourage their involvement?*

*Did you feel the team was equipped to do this or did you experience any challenges?*

*Is there anything about this that you would do differently/do differently now?*

**Please could you tell me what you remember about how the PRO information was reported following the trial?** **[or general experience of reporting PRO findings?]**

*Do you remember if there was a plan for the dissemination of results?*

*Did you feel the team was equipped to do this or did you experience any challenges?*

*Is there anything about this that you would do differently/do differently now?*

**Are you familiar with any guidelines or training that are specifically intended to assist when including PRO information in protocols?**

*If yes, please could you tell me more about them?*

*If no, do you think guidelines or training would be helpful? How could they be implemented?*

**Have you ever received any PRO-specific training?**

*If yes, please could you tell me more about the training you have received?*

*What was good about the training? What was bad?*

*Can you think of any examples of particularly effective/ineffective PRO-specific training you have received?*

*If no, do you think PRO-specific training would have been helpful?*

*What prevented you from receiving training?*

**Have you got any experience of providing training yourself?**

*If yes, please could you tell me about it?*

*What do you think are the key components of PRO training?*

**Are you familiar with any online PRO-training resources?**

*If yes, please could you tell me a little about them?*

*If no, do you think these would be helpful to your or your colleagues?*

**Are you familiar with CONSORT PRO reporting guidelines?**

*How do you think these guidelines can be made more accessible?*

*Do you think these guidelines could be better implemented? How?*

**Finally, would you say you are optimistic about the collection and reporting of PROs in cancer clinical trials in future?**

*What are the next steps?*

**OPTIONAL QUESTIONS**

1. **For participants in senior research positions/journal editors**

**What do you think are the barriers/enablers for offering PRO-specific training to researchers who are working on trials/associate editors and peer reviewers?**

*Have you experienced any of these challenges in your own work?*

*In your experience, how could this kind of training be promoted?*

1. **For participants with experience of being a patient participant in research or improving patient engagement**

**What do you think can be done to encourage patients/participants to take part in the PRO component of a trial?**

*What are the challenges/motivators?*

*What would encourage more co-design?*

*Have you seen PRO results as a patient/used PRO data in treatment decisions? Where?*

**Are there any other aspects of PROs in clinical cancer trials that you would like to discuss?**

**Thank you for taking part.**

*Telephone interview topic guide – Patient participant interviewees*

**Introduction**

1. Re-state purpose of the interview: *Discuss your experience and understanding of Patient-Reported Outcomes in clinical cancer trials.*
2. Any questions or clarifications before beginning? If appropriate, have participants’ scoring information, protocol or publication for reference.

**Topics to be covered:**

**Please could you tell me a little about your background and your experience of cancer research?**

[Consider which of the questions are of most relevance to participant and their expertise but cover all topics]

**[*Give short outline of PROs in cancer clinical trials*]**

**What do you think can be done to encourage patients/participants to take part in the PRO component of a trial?**

*What are the challenges/motivators?*

*What about encouraging patients/participants from minority backgrounds?*

**What can be done to encourage people who have experienced cancer to become more involved in the co-design of trials?**

*What are the challenges/motivators?*

**What were your sources of information during the time around your diagnosis and afterwards?**

*What was good about these?*

*How could these have been improved?*

**Did you ever use PRO data to inform your decision-making?**

*Were PRO data ever used in treatment decisions?*

*Do you think this kind of information would be helpful?*

**Have you ever seen PRO results as a patient?**

*Do you think receiving this kind of information during the course of your treatment would be helpful?*

*If so/not, why?*

**Since you became involved in cancer research, have you received any training about PROs?**

*If so, please could you tell me about it? What was good/bad?*

*Can you think of any examples of particularly effective/ineffective PRO-specific training you have received?*

*Do you think this would be helpful for you and others?*

*How do you think this would be best provided/accessed?*

**Are there any other aspects of PROs in clinical cancer trials that you would like to discuss?**

**Thank you for taking part.**

*Telephone interview topic guide – funding panellists*

**Introduction**

1. Re-state purpose of the interview: *Discuss your experience and understanding of Patient-Reported Outcomes in clinical cancer trials.*
2. Any questions or clarifications before beginning? If appropriate, have participants’ scoring information, protocol or publication for reference.

**Topics to be covered:**

**Please could you tell me a little about your background and your experience of being on a funding review panel?**

[Consider which of the questions are of most relevance to participant and their expertise but cover all topics]

**Have you had any experience or contact with PROs in your work/research?**

*How did it come about? What was your role within this work?*

*If not, why? Is it likely you will in future?*

**In your role as a panellist, do you often come across PRO content in grant applications?**

*Do you have guidance from the funding body on how best to assess PRO content in an application?*

*Is it usual for you to receive guidance from the funding body on how to best assess content related to other outcomes?*

**Have you ever received any PRO-specific training in your capacity as a funding panellist or in your other roles?**

*If yes, please could you tell me more about the training you have received?*

*What was good about the training? What was bad?*

*Can you think of any examples of particularly effective/ineffective PRO-specific training you have received?*

*If no, do you think PRO-specific training would be helpful* *for you and your panellist colleagues?*

*What prevented you from receiving training? How would it best reach you and your colleagues?*

**Are you aware of any information resources you could use to get PRO-related guidance?**

*If yes, please could you tell me more about them?*

*If no, do you think this would be helpful? How could they be implemented?*

*Does your funding body endorse any methodological guidelines? If not, why? Would this be helpful for you and your panellist colleagues?*

**Does the funding panel consider whether a study will report each of their outcomes?**

*Following completion of a trial, does the panel review whether each outcome has been reported/published?*

*What are the barriers for reporting? How might it be encouraged?*

*Is there a role for funders in checking this?*

**Are there any other aspects of PROs in clinical cancer trials that you would like to discuss?**

**Thank you for taking part.**

**Supplementary Appendix S3: PRO-related information needs**

| **Time period and sub-theme** | **Sample quote and source and primary stakeholder group** |
| --- | --- |
| 1. Diagnosis | |
| Variation in information needs | “There’s a bit of being in denial that it’s happening, or they are protecting themselves, you know some people really don’t want to know the prognosis, really don’t want to know the statistics, my personality and my brain couldn’t get enough of it, I just really couldn’t get enough of it, but I know that other people are different to me and they absolutely just do what the doctor tells them.” [025, Lived Experience] |
| Providing information to “shell-shocked” individuals | “But the other thing you see is that I thought I was handling it pretty well but I was under such a lot of stress … I did not take it in very well at all.” [036, Lived Experience] |
| Early introduction of PRO-related information | “I think that quality of life information should come from the beginning. It’s important to consider that. Independently, that the patient at the beginning of course will be in shock by their diagnosis and they will try to survive at any price. But if they understand that someone cares about quality of life, they will feel also better to talk about symptoms and to talk about adverse effect because what happens is that patients not always report adverse effects, particularly those related with pain or those symptoms that affect dramatically their daily activity because they are scared to be not to be allowed to continue the treatment. So you need to create the confidence and the proper dialogue, accurate dialogue with the medical team to talk about this. And that’s why these talks should appear at the beginning even if the patient does not ask.” [038, Lived Experience] |
| Patients’ awareness of what is PRO data | “I suppose the only thing is about getting members of the public to understand what we mean by health-related quality of life. Everything that comes under that, to do with their global wellbeing, I think there’s a big educative piece of work to be done there as well. Health-related quality of life is quite a new area. It’s only been around since the 1980s or 1990s. It’s all very much in its infancy but I think there’s quite a big piece of work to be done around educating the public in what we mean by it and why it’s important for them to have conversations with their clinicians.” [037, Journal Editor/Funder/Regulator] |
| 1. Information seeking from non-clinical sources | |
| Insufficient detail | “It was very noticeable the majority of leaflets, if they were there, were from charities and were very patchy in what you could get and also in something like my blood cancer, blood cancers generally – and we looked at this subject – because there are so many different types and everything else, although luckily I’ve got something that’s very common, but you know what I mean, so there was more information, if you’ve got one of the rarer ones, the chances of you finding the right leaflet at the moment you need it would be actually nil” [026, Lived Experience] |
| Dubious sources and lack of quality control | “He claimed that her changing her diet and beetroot juice had got rid of this stage four lung cancer. And then I said “had she had chemo or radiotherapy?”, “oh yes, both but it’s the beetroot juice that did the trick”… this woman self-published a book that people will be reading and a lot of the online stuff … the evidence isn’t there and … it could be dangerous. So, people need to have trusted sources of information to go to.” [033, Lived Experience] |
| Influence of non-clinical information sources | “The popular journalists … they are so influential on people’s thoughts… Because some of them use a headline that will, ‘ching’, that will completely change everybody’s view on a drug, almost overnight and balancing those …They have a bigger influence than the academic on the average person.” [026, Lived Experience] |
| Accessibility of non-clinical information sources | “I just found it extremely useful and as long as it was fairly, as long as the language was reasonably straightforward and the descriptions were not too technical and there were links to go more detailed information if you wanted it … And to me it’s just very important to bear in mind the audience that you’re talking to in the information that you’re trying to get across.” [032, Lived Experience] |
| Availability of PRO-related data from non-clinical information sources | “When you go to scientific conferences or whatever else, PRO’s are always the last thing that’s mentioned and always mentioned in a very statistical formulation without any real personality of character attached to them and yet patient to patient, if you spend your time talking to somebody, these are the things that they are really talking about, I mean obviously they all want, everybody would like to have a cure and great outcome but a lot of the time they are talking about side effects or you know, the reality of taking a drug, not the outcome of taking a drug.” [026, Lived Experience] |
| Poor access to peer review journals | “Some people online still have paywalls, so even if someone publishes an interesting study the people who need to know about it, which is patient groups, can't get at it because some bloody journal publisher has decided that knowledge needs to be kept secret. Even though every patient on that trial volunteered to help humanity, the journal publishers have decided that no, it must be kept secret.” [007, Lived Experience] |
| Perceived competence in seeking information | “I am reasonably confident that I know how to find good information on the internet and therefore what I was looking for was real… the sort of proper medical output information like from WebMD and sites like this, that I felt were giving proper summaries of what the current state of knowledge was and how that might be applied to an individual’s case.” [032, Lived Experience] |
| 1. Information seeking from clinical sources | |
| Perception that clinicians are not forthcoming with information | “With my daughter and her brain tumour diagnosis. Unless you ask a specific question, they don’t give you information but you don’t know what the questions are that you need to ask [laughter] because you’re new. It’s all new on the journey. It’s a steep learning curve.” [037, Journal Editor/Funder/Regulator] |
| Need to generate more data to inform treatment decisions | “They do use treatments but they use treatments which have not been developed for this condition precisely. In the case of my daughter … she received the standard chemotherapy and there is really no sufficient data about long term risks with NF patients who receive these kind of treatments” [039, Journal Editor/Funder/Regulator] |
| Perception that clinicians are dismissive of requests for PRO related information | “I had already asked about side-effects and so on and these were dismissed by a wave of the hand” [036, Lived Experience] |
| Implicit trust in clinicians | “I had cancer, that’s the choice and I made the choice quite easily, with my consultants and everything informing me of that was the best way and you trust them you know, you trust them to know what they’re doing” [025, Lived Experience] |
| Provision and use of PRO data | “When I had the amputation, the way they looked at making that decision because a different surgeon had offered an alternative approach which would have saved the leg even though it would probably have made me just as disabled as if I had lost the leg. When we looked at those two alternatives I suppose we took account of the fact that people that had had a similar amputation did lead a full and active life. But it wasn’t structured information, it was much more looking for the case examples.” [027, Lived Experience] |
| 1. Choosing treatments | |
| Selective disclosure of information by clinicians | “In some cases, we have discussed with clinicians or with researchers why these symptoms are not mentioned to patients, they explain “okay, but who will tell me which patients will develop symptoms and which patients won’t develop that much? So why would I prefer them to be super scared about symptoms if they probably won’t develop all of them?” [037, Journal Editor/Funder/Regulator] |
| Awareness of patient preference and shared decision-making | “The individual patient preference is important because some people will undergo anything for a very short period of prolonged survival, and other people feel very strongly that quality of life not quantity matters more, and you have to be certain that you know what that patient wants. And I don't think eliciting that patient's priorities and goals is taken as seriously as it should be.” [030, Journal Editor/Funder/Regulator] |
| Imbalanced power dynamic between patient and clinician | “The imbalance in power and information is massive … particularly oncology. The doctors have all your test results and your scan results and they have an upfront discussion about you and they pretty well decide what's going to happen to you. You may then rock up at a clinic and you have to take on board both a diagnosis, what the scan shows, what a multi-disciplinary team has suggested is the right way forward, and you sit there in total shellshock and say 'oh, well doctor if you think that's the right thing'. I mean people cannot make shared decisions in a state of shellshock, they need time. “ [030, Journal Editor/Funder/Regulator] |
| Clinician’s focus on survival | “When you’ve got a short day and a lot of patients and limited things you can do you’ve got to prioritise haven’t you and if you’re responsible for the survival of patients… I think for a lot of haematologists as well that the focus is very, very much on is this patient going to live or die, for a lot of cancer clinicians altogether not just leukaemia and haematology but for all cancers. The big, big issue that doctors have to deal with, as opposed to nurses, and for the doctors it’s always going to be survival of the patient that comes in way above everything else.” [014, Trialist/Chief Investigator] |
| Transparency about treatment realities | “If [oncologists] were more honest about … the limitations of data in terms of what it will actually bring to patients, I think more patients would welcome that, but I think there is a reluctance among clinicians to face the uncomfortable truth that none of the treatment options available to them may actually make a difference and it may be more appropriate to say that, or that the extra month or the extra six months won't be gained at better quality life and would you prefer to go to Australia and say goodbye to your brother or spend the last six months coming along and getting toxic treatment in my department and feeling dreadful. I just think we don't have enough honest conversations.” [030, Journal Editor/Funder/Regulator] |
| Emphasis on “fight with cancer” undermining discussions of treatments | I remember having a conversation with somebody who was a doctor about some of these diseases in this group that I’ve got, and him saying that really, what he wanted to tell the patient was you know, “don’t worry about the treatment, go away and enjoy the 18 months you’ve got and don’t put yourself through this”. And I said, “Well why can’t you do that?” and he said, “Well, they sort of want to battle and I want to battle and none of us in the end are really brave enough to talk about it” … it’s the way we portray cancer” [026, Lived Experience] |
| Informed consent | “[Patients] feel that more indicators should be measured and that clinicians should assess not only the disease activity and progression but should assess the adverse effects and the daily function... providing also different strategies to include this functioning and to manage the adverse effect. And also to understand better which symptoms will disappear when treatment is stopped and what kind of adverse effect will remain longer. Because, also the capability or the willingness for patients to accept worse effects will depend on how long this will remain in their life. So they will wait also or they would try to make a balanced amongst benefit, risk and also the time they will need to sacrifice their quality of life.” [038, Lived Experience] |
| Treatment choices based on non-clinical information sources | “I was diagnosed with breast cancer… I decided though initially I was going to “do the works”... I was going to do everything but then … I started to read up about what I was in for, try and get some information of NHS and proper websites, not your Internet sometimes rubbish. And I had already asked about side-effects and so on and these were dismissed by a wave of the hand and the more I looked, the more I realised that I could be in for some side-effects I wasn’t really, I was really not happy to potentially have such as I have asthma and I was looking forward apparently to losing quite a bit of lung function with the regimen they were prescribing. Anyway, I ended up not having any treatment other than surgery, which was fine.” [032, Lived Experience] |
| Availability of treatment options | “A lot of doctors focus on survival. But there are the two key questions for patients aren’t there? There’s how long am I going to live and what’s my life going to be like in the meantime so in other words survival and quality of life are the two primary end points for the majority of patients. But I think that because doctors haven’t got any direct way of addressing the quality of life end points they tend to focus more on the survival” [014, Trialist/Chief Investigator] |
| Clinicians minimising side-effects | “But it’s very important, particularly on some of the newer treatments where the belief is that some of the side effects are being under-reported and what seems acceptable to a clinician, “oh well, we’ve buggered up their thyroid but that’s alright, we can medicate against that”. Or “well, they’ve got a grade three skin reaction but that’s ok”. I mean, people wouldn’t leave the house, or “oh well, it’s given you grade four diarrhoea but never mind, you’re still alive”. It’s like well, people have a different attitude if they’re the person experiencing that.” [033. Lived Experience] |
| Variation in clinician interest in PROs | “Ten years ago, I thought we’d publish these studies and everybody would say, ‘Yeah, this is really important’ but actually, it’s not a priority for a lot of clinical groups. In my own discipline, there are a few exceptional clinicians who refer huge numbers of patients but the clinician next door, seeing exactly the same type of patients, refers none. I think that’s been the experience of everyone working in the Survivorship field; that if a patient is very lucky, he’ll see someone who’s interested and they might then get an appropriate referral to someone who knows what they’re talking about but otherwise, it doesn’t happen.” [020, Trialist/Chief Investigator] |
| Encouraging provision of PRO information to inform decisions | “I think it would be helpful and as a potential patient or family member who might be a patient I would really like that information available to me in making decisions about treatment. Even if the results of the study show there’s no difference between treatment X and treatment Y I’d want to know that, and not assumed it’s based on clinical outcomes and toxicity data” [005, International Expert] |
| 1. Receiving treatment | |
| Patients underreporting side-effects | “There does seem to be either an under reporting of the side effects or a patient’s not wishing to waste the clinicians time when they only have a consultation every few months, with side effects that are, at the time of the consultation, no longer troubling them but they have troubled them in the interim.” [033, Lived Experience] |
| Change in information needs as treatment progresses | “As time goes on, you have your initial treatment and then obviously other things crop up and I think that’s when you need more information. It’s not, if you don’t have this, you will die, it’s if you don’t have this, this might happen.” [025, Lived Experience] |
| Patients seeking validation of their experiences from other patients rather than clinicians | “You start to see on websites, people are saying, “anybody else experience this?”, and then a flurry of responses come in, “oh yeah, I’m still having pain eight years on, I’m still having pain two years on” … I bet none of this is reported or very little and yet within the help groups people are, so, I think one of the things is making it ok for people to report stuff that they might consider trivial if they were going to the doctor.” [033, Lived Experience] |
| Patients seeking validation of their experiences from clinicians | “I think what I wanted was reassurance that whatever was happening to me wasn’t completely out there, you know, they’d seen it before, they’d done it before, it wasn’t beyond the realms of normal for that treatment. I: Did you get that reassurance from your clinicians? IV: Yes, absolutely, yeah.” [025, Lived Experience] |
| Role of nurses in eliciting PRO related information from patients during treatment | “it’s interesting how varied doctors are and I think nurses absolutely understand that quality of life is immensely important to patients” [014, Trialist/Chief Investigator] |
| Time-related challenges to PRO-centred clinical interactions | “Patients don’t always recognise that the consequence of treatment is a consequence of treatment because it starts three years later or five years later. They find it very difficult to see the connection sometimes … empowering the patient and educating the patient for every possibility is a really very difficult thing to do in a cash-strapped, busy Oncology Unit with a limited time to follow-up when the priority is about whether the cancer recurred, rather than what are the symptoms the patients are getting.” [020, Trialist/Chief Investigator] |
| 1. Late effects | |
| Regret about receiving insufficient information to make treatment decisions | “I’ve had male patients who now say they wish they hadn’t had radical treatments for prostate cancer, because of the, you know, what it’s left them with, incontinence, impotence. Breast cancer patients, years on who have got lymphedema and can’t lift their arms up and had debilitating things like that or weight gain from steroids. So, there are various side effects of treatments that in a clinical setting are deemed acceptable but when you’re living with that day on day or inflammational pain that can be quite difficult.” [033, Lived Experience] |

**Supplementary Appendix S4: Generation of useful data through PROs**

|  | **Themes and sub-themes** | **Sample quote and source and primary stakeholder group** |
| --- | --- | --- |
| Facilitators | Prominence of PPI in funding applications | “When you put in a grant application nowadays rather than the token gesture of, 'Oh yes, we'll have a service user on our steering group,' you have to say so much more than that. You have to involve people right from the inception of your ideas, really, and all the way through and that's what we need and we need more of that.” [021, Trilaist/Chief Investigator] |
|  | Expectation of PPI in study development | “On the [funding] Board, the question that’s always asked is, ‘is this outcome meaningful for patients?’ It’s a pragmatic funding board and sometimes, mortality might not even be the right outcome. Patients can say, ‘we don’t care about that, we care about something else.’ With the outcome choice, there’s always a question – ‘How meaningful is this for patients?’ If applicants don’t give a narrative around why they’ve chosen that, in terms of a patient voice, then we often say, ‘Do you think you could choose an outcome which is more meaningful and relevant for patients?’” [042. Journal Editor/Funder/Regulator] |
|  | Prioritisation of patient perspective | “Nobody but the patient can tell us what the patient is experiencing, Clinicians have a great deal of experience in treating their patients in areas of speciality, but they’re not the one that’s experiencing it, and I think that with a lot of the more sort of regulatory and the scientific emphasis on patient-centeredness in recent years, everybody is recognising more that we really need to be directly engaged with the patients in a very…first hand kind of way.” [010, International Expert] |
|  | Availability of PPI | “I've been asked often by clinical researchers, 'Where do we find these patients?' and my answer every time has been, 'Have you opened the door to your clinic lately?' That's where they are.' There are plenty now of patient groups. There are plenty of involved patients.” [007, Lived Experience] |
|  | Emphasising value of patients’ contribution | “Patients give you a 360 degree viewpoint of what's going on. Without that you get less.” [034. Lived Experience] |
|  | Increased patient centricity through personal experience | “There has been a good push in oncology and a lot of that is because people suffer and they watch their family members suffer and they say, yeah, this information is important. I wish I had known it, so now I’m going to go and try to roll it into my studies.” [011, International Expert] |
|  | PPI and research impact | “One of the barriers… to impact is because you never really showed that the outcomes in your trial were really what patients wanted. As we get better at using PPI to understand the research, using patients and the public to drive the research agenda, we will understand better that the end points we’re using in trials, whilst they may be the most valid are not necessarily what we need to prove to truly change practice.” [018, Trialist/Chief Investigator] |
| Barriers | Misunderstanding PPI | “Industry is very worried about this …they’re quite shocked that they’ve got to do this and they keep saying, ‘How should we best do it?’ I said to them on in the first place, ‘One of the things you can very simply do is work with a small group of patients on how you write this up’. They said, ‘Oh my god. There isn’t time to recruit patients. We haven’t got the time’... A lot of industry really, really struggle with PPI and they think we are just talking about recruitment, rather than advisory capacity. A lot of them just don't get it at all or they think it's illegal for them to talk to patients.” [041, Trialist/Chief Investigator] |
|  | Prognosis-related challenges to PPI | “The colleague I joined on the [patient] group, he’d lost his wife, so he was a carer rather than a direct patient. And I replaced somebody how had lost a husband because part of the issue with lung cancer is there have been so very few survivors and those that are interested in sitting what could be seen as quite turgid committees really, with looking at lots of paperwork and academia.” [033, Lived Experience] |
|  | Difficulty recruiting to PPI | “I became chair of [a patient group] and we had … about 24 members of the group. But theoretically we should have numbered something close to 50 because there were 23 [patient] groups and we had the funding and the brief to put two people on to each one of them. Now finding patients for some of these groups has been incredibly difficult over the years, this is patients and carers by the way not just patients.” [027, Lived Experience] |
|  | Failing to remunerate PPI input | “If you're going to seriously get some trained patient advocates … you'd probably have to pay them rather like whoever else is around the table who is getting paid…I think to continually feel that the expertise of patients can be tapped into without any concern to remunerate them for their time and effort is wrong… Patients being asked to do things and everyone else sitting round the table is getting paid to be there and they're not? Yes, happens all the time and if you talk to the seasoned patient advocates it makes them furious.” [030, Journal Editor/Funder/Regulator] |
|  | Patients outnumbered in research environment | “So on the [research group], the eighteen, what I call, “medics”, you know, doctors, professors … and then two lay people. Well, already you’re on the back foot aren’t you if there’s only two of you.” [025, Lived Experience] |
|  | Researcher reluctance towards PPI | “I perhaps found it more so with older [researchers] than the younger ones who are coming through the system now. But certainly there are many who see the whole sort of PPI and taking too much notice of what the patients’ want and the patients’ opinions as a bit of an intrusion on the quality of the research and I’ve definitely heard these sorts of opinions expressed.” [032, Lived Experience] |
|  | Representativeness of PPI | “Within patient participation, you need a massive broad section of people but that’s hard … ‘we need five people in each group but you need someone who is young, you need someone who is old, we need someone who is poor, we need someone who is rich, somebody who is black, somebody who is white’, we need a real cross section isn’t it? And I think with two people in each group, you can’t possibly have that.” [025, Lived Experience] |
|  | Ensuring PPI is meaningful | “I think we do pay a bit of lip service to. 'Oh yes, I've had all this user involvement,' but how involved are they? Are they equal partners? Because often they're not.” [021, Trialists/Chief Investigators] |
|  | Including PPI at too late a stage | “I work with quite a lot of Clinical Trials Units, helping them to make sure that there is patient engagement and involvement. It’s like fairy dust through trial design. I get tired of just being asked to comment on Patient Information Sheets. By then, it’s too late.” [037, Journal Editor/Funder/Regulator] |
|  | Differing views between researchers and patients | “I think traditionally, a lot of clinical research is done by physicians and I think, traditionally, unfortunately, many patients felt, ‘oh, the physician knows best’. Clearly, as time moved on and trials were done and the outcomes were relevant to researchers. Perhaps far less relevant to outcomes that patients felt more important.” [031, Journal Editor/Funder/Regulator] |
|  |  |  |
| Facilitators | PROs reflecting patients’ interests | “When I get together with a [patient] group, [PROs] are the things they are really talking about a lot of the time…” [026, Lived Experience] |
|  | Including PPI at a stage when they can select outcomes | “You have to start right at the beginning and talk about a collaborative, a proper collaborative relationship between the PPI people, between patients and the researchers and to try to get everybody to see that there are benefits to all of us from having patient involvement working in a properly balanced, effective way in the whole system… Whatever this relationship is between the patient and the researcher and working on outcomes, it has to start very early and it has to be based on a mutual understanding of ‘we’re working on this together’.” [032. Lived Experience] |
|  | PPI groups as PRO proponents | “In cancer, particularly, I do see that the PROs tend to get relegated to extra information but not always and I do think many patient groups are pushing them forward.” [011, International Expert] |
|  | Investment in patients as PPI proponents | “I think if it’s framed in the right way so that the patients involved can see how meaningful this is for them then it seems natural to me that it should play an important part and that they would see that. Because this is about them, it’s not about whether let’s say a particular drug is effective on a particular cancer, it’s about the whole breadth of “what was it like for me?” as well.” [032, Lived Experience] |
|  | PPI involvement conducive to more PRO use | “In my role on the [patient groups], I wouldn’t sanction a trial, I wouldn’t endorse it if it didn’t have some element of PRO in it.” [037, Journal Editor/Funder/Regulator] |
| Barriers | Need to identify PPI individuals to take a personal interest in PROs | “You almost need a PRO advocate within the patient community … Because not everyone’s going to be that interested in it... they’ll be too busy looking into whatever is their particular interest within the cancer realm – which is huge – so maybe you need to have just some people who are really good at it who can then be the PRO expert for that community.” [025, Lived Experience] |
| Facilitators | Shifted emphasis from survival to quality of survival for patients | “[Researchers] seem to think it’s a breakthrough if a particular treatment shows promise and somebody’s life is extended for three or four weeks. For me, that’s not a satisfactory outcome. I think they could listen to patients more and actually and if they understood that often what patients want is not necessarily an extended overall survival but a better quality of life in that progression-free period.” [037, Journal Editor/Funder/Regulator] |
|  | Shifted emphasis from survival to quality of survival with availability of more treatment options | “When we were looking at chemotherapies that made people absolutely miserable, it was worth it to save their lives but now the treatment has advanced to a point where is not just a matter of, can we keep you alive, but, can we keep you alive and comfortable, can we improve your survival and improve your symptoms, then it starts becoming more important to actually be able to assess the symptoms as opposed to when we have drugs that just, universally made people feel God awful.” [010, International Expert] |
|  | Different areas of cancer research requiring different trial outcomes | “For some cancers, measuring survival is going to be the most important outcome to look at, so, patient reported outcomes are maybe not as important, but a lot of the work we do is related to palliative care for people with cancer, so the patient reported outcomes are like, crucial in that sort of study, but I think it depends very much on what is the clinical question and what is the treatment that is being evaluated, what’s it trying to do.” [015, Trialist/Chief Investigator] |
|  | PROs and research impact | “There is a big focus from funders and big focus in the universities now on Impact Assessments and I think that’s going to really drive a big interest in PROs because clearly, if you can demonstrate that not only do patients live longer but in real life, they actually have a better quality of life. Their own perception of the outcome is better. That’s a very powerful statement about impact because it’s virtually impossible to see if somebody lived longer in that real life setting and so these tools could then become really useful there.” [018, Trialist/Chief Investigator] |
|  | Changing research needs in cancer | “I think the whole initiative and recognition that if one in two people are going to have cancer in future, and the aim is that 75% will survive 10 years or more, then that’s going to be an awful lot more people coming in to this pool of “living with and beyond”… there is a whole load of difference between somebody who has a melanoma removed or somebody who has an early stage breast cancer removed and somebody who has pancreatic cancer, there’s just a world of difference there.” [033, Lived Experience] |
| Barriers | Gap in research using PROs where survival is low | “Unfortunately lung cancer has not had good prognoses …Which has meant that studies have been very scarce and equally an awful lot of the patients have had their lungs damaged from smoking or other substances which then means they’re incredibly poorly and they haven’t, often, been able to get traction in study numbers with long enough survival so, it’s always been a sort of post-mortem type study and length to recurrence or death, seems to have been an outcome rather than patient reported outcomes.” [033, Lived Experience] |
|  | Gap in research including PROs in uncommon cancers | “The time I was first diagnosed, I don’t think quality of life took a significant part in cancer studies, certainly not in the rarer tumours, it probably had started, well it had started in more common tumours.” [027, Lived Experience] |
|  | Gap between PRO researchers and other researchers | “I started to deal with brain tumour patients who had been treated with radiation therapy years ago and seeing that some of these patients had serious cognitive problems. And these patients survived. So, colleagues asked, ‘why bother? These patients are still alive, they had a brain tumour but they hadn’t died. So, why are you bothering with the problems that they have?’ And I thought, ‘well, these patients do have problems, and it’s not just that they’re surviving but their life has changed… We have to move this forward because we really don’t know what we’re actually doing. We are trying to keep these patients alive but is it a good idea, if these patients are that much damaged?’” [023, International Expert] |
|  | Inclusion of PROs on obligatory basis | “The majority of oncologists who are doing clinical trials are not really very interested in patient reported outcomes. They’re interested in survival and …they are doing some patient recorded outcomes … because there has been a very slow movement to say that you can’t report things without this. It’s not a priority and certainly, if it’s not the primary focus of the trial, perhaps it doesn’t get as much thought or attention.” [020, Trialist/Chief Investigator] |
|  | Research priorities not conducive to including PROs | “I quite like [charity’s] research policy, which is actually focusing on these cancers on a league but, in so doing, they now regard patient-reported outcomes as even less important than it was before. The [national funding body] is more interested in funding things that save money in the NHS. So I think one of the problems is that funders are not funding this research as an issue of importance.” [007, Lived Experience] |
|  | Continued emphasis upon survival | “Oncology trials centre on reduction in patients who are sick, it’s about reduction in survival. Patients are not necessarily expected to get much better. It’s about reducing the time for progression or death. It’s because, what is distinctive about oncology, especially in later or advanced cancer, it’s about longevity and that’s what ranks. That’s the subject-matter content. It’s not about wellbeing, functioning, symptomatology, …of course safety and tolerability are very important … But when it comes to on the efficacy side, it’s about survival, because of the nature of the disease … What is said to be the most meaningful outcome are those time to event outcomes. That’s what the experts and other stakeholders say.” [029, International Expert] |
| Facilitators | Using PPI to design PRO design | “Encouraging patient and public involvement … from the very beginning, in terms of developing what the patient-reported outcome measures are. In terms of, is the trial design feasible for the participants? Are they likely to carry on with a trial or drop out early because it places too much of a burden on them, for instance?” [041, Trialist/Chief Investigator] |
|  | Permitting the use of proxies to complete PROMs | “Well so the very poorly patients you could always say that it’s okay for somebody else to fill this in for you if you don’t feel like actually writing it out yourself, you can have somebody with you and give them your answers so they can write them down for you. You could make it clear that it’s perfectly valid and okay for someone else to actually fill it in for you, physically to fill in the answers on the piece of paper and lick on the stamp and send it off.” [014, Trialist/Chief Investigator] |
|  | Participants feeling empowered through PROM completion | “If [PROMs are] very much are relevant to the patient then patients can feel a little bit empowered by sharing their very personalised feelings about things or how the outcomes of their whatever they’re trying or whatever drugs or therapies or whatever they’re trying are recorded they can feel good about that.” [008, International Expert] |
|  | Inclusion of patients in the development of PROMs | “When you get patients who are more involved in participation and involved in the decision making when it comes to creating the questionnaire rather than just working out how you answer the questions, you get a greater amount of participation from the patient” [034, Lived Experience] |
|  | Balancing research aims and participant burden when selecting PROMs | “The Patient Reported Outcomes should count. Perhaps one generic measure, one disease-specific measure and an excess of them may [put off] patients who are already sick or even those who are not very sick. So we have to be mindful that when considered, Patient Reported Outcomes need to be judiciously planned in order to avoid patient burden, which could compromise the integrity and validity of the results.” [029, International Expert] |
|  | Adjusting the demands of the PROM to suit participants | “Maybe you have it at different tiers … if a patient’s feeling good one day, he wants to report a lot of things, if he’s not feeling so good or he’s busy or doing things he only wants to report on certain things … you could make it very easy for people is that if they’re feeling okay they might just want to press a button to say yeah, I feel the same as yesterday but, you know, and it replicates that feeling or if they’ve got time and they want to go to, further and open up some new boxes for them where they can go into more detail on what they’re feeling.” [003, Lived Experience] |
|  | Streamlining PRO delivery | “We’ve been a champion … for using many single item measures and really… integrating it into the clinical trial process. Administering only the tools that you absolutely need at the time points that are scientifically justified and really trying to give sites the flexibility to make it work in their workflow while still maintaining a high amount of consistency across sites … those are kind of all the things I mean by a ‘minimalist approach.’” [022, International Expert] |
|  | Use of electronic PROMs | “The new ePRO approach … 50% of our measures… are transformed into electronic measures straightaway. What you’re seeing is people are starting to collect data electronically. It’s usually easier, faster and the compliance is better.” [002,. International Expert] |
|  | Offering a range of PROM options to suit participants | “A very, very small number of men completed it electronically. Almost all of them preferred paper. And they are men… and they were 50-69 years of age ten years ago. So they were men in the older age group. A small number filled them in electronically, particularly if they’d moved abroad but not very many. We have continued to offer them electronic completion but not many of them have wanted to do it.” [028, Trialist/Chief Investigator] |
|  | Clarifying link between PROM completion and treatment | “It’s just reiterating the importance of filling in the details … if you put down that you’re not feeling great or you’re not sick, it doesn’t necessarily mean you’re going to be taken off the medicine.” [006, Trialist/Chief Investigator] |
|  | Ensuring participants are informed of why PROMs are completed and what happens with data | “Clear education pieces about … why specifically is PRO data being collected, why is it important and why is it important that if we don’t have the full dataset that the data becomes difficult to interpret and we can’t draw any conclusions. So, that kind of reemphasises the importance of engagement in collecting the PRO data overall.” [006, Trialist/Chief Investigator] |
|  | Supporting participants to complete PROM | “It’s higher for some questionnaires than others but pretty consistently over 85% response at every time point through to six years… We had nurses who encouraged the men to complete the questionnaires in the clinical centres. I’m sure that made a difference. The questionnaires were sent out by post but we had Study Nurses who the men mostly saw each year and they encouraged them to complete them, particularly as it was such a long questionnaire. We had a lot of complaints about the length of the questionnaire but they still rather gamely did it, which was just amazing.” [028, Trialist/Chief Investigator] |
| Barriers | PROMs appearing irrelevant to patients | “I don’t think patients see the link between what they’re filling in on paper and what they want to know about their treatment. Quite often, you get from a patient, ‘Yeah, I filled out the form. Most of those boxes didn’t apply to me. It was asking about my pain but I don’t have pain’ or ‘It was asking about my sex life and that’s not relevant to me’ or whatever. So I think, very often, patients don’t really understand… why they’re filling out the forms” [018, Trialist/Chief Investigator] |
|  | Poor participant engagement resulting in missing data | “It’s very patchy, you can get back some questionnaires from some patients and don’t get other ones back from the same patients, patients drop out all together and don’t bother to return quality of life questionnaires after the first one or two. So must be familiar with that, missing data, it’s a huge hurdle to quality of life analysis. I think that it is regarded as a very secondary end point by a lot of people including patients.” [014, Trialist/Chief Investigator] |
|  | PROMs designed with different aims than those of patients | “I get the impression that a lot of these forms are developed to suit a different end and they’re actually driven by the needs of the trial, rather than by the needs of the patient group…If you look at some of the tools, you think, ‘Why are they asking that?’ Actually, they’re asking that because it’s going to be a measure where it’s going to have a higher chance of showing a difference, rather than actually measuring what patients want you to be able to demonstrate.” [018, Trialist/Chief Investigator] |
|  | Practical challenges of electronic PROMs | “Partly funding, partly technology. It's expensive to do an app … There may be apps out there that are ready made or whatever and I've been contacted by companies that say, 'Use this app for ...' or, 'We do apps and computer systems for collection of data in trials,' and stuff like that and I'm sure they've got those but it does cost money and our internal clinical trials units tend to have a real problem with programming… On the PRO side of things, of course, if you're going to do that there's all that data security issues and encryption, etc, etc, that make it harder as well.” [017, Trialist/Chief Investigator] |
|  | Participants not seeing contribution of PROMs to cancer research | “I'm not convinced that [PROMs] are helpful in every case. I am quite convinced that they should be useful, that they could be useful but I'm not convinced that we're doing the right ones in the right way at the right time for the right reasons because we're still doing stuff that we've been doing for 13 years and no one's taking a blind bit of notice of it and life has changed.” [007, Lived Experience] |
|  | False compliance | “The number of people that have told me they blatantly lie is astonishing! Because they don’t see why it matters, they get nothing back, it’s never mentioned” [043. Trialist/Chief Investigator] |
|  | Failure to link PROM completion to care | “There’s an implicit trust that if something’s wrong in that blood sample the medical team will be dealing with it … they know it’s been used for their care and they know that if something’s wrong, they will be told. Here the medical team aren’t, they’re kind of collecting [PROMs] back in and going, ‘oh thank you very much’ but they know they’re not being used because we know the medical team don’t really see the data in time for it to be useful … patients pick up on that.” [043, Trialist/Chief Investigator] |
|  | Participant engagement and prognosis | “I was in a really bad place at the time where I had a 5% chance of survival but they did work on a cure for me rather than palliative care … as soon as they said, ‘Right, we want to cure you. We’ll work on a cure for you’, it was there. Within those trials I think you have to allow for if they’re not going the right way … if I saw the figures going down the wrong way and it was not looking good would I actually want to report those on a daily basis? I’m not sure.” [003, Lived Experience] |
| Facilitators | Modifying PROMs to suit research aims and population | “We reviewed the instrument as a team and we looked at all the available questionnaires. Having done pilot studies in this patient group and a number of interventional studies using this questionnaire and others, we felt that, at the time, that this modified version was probably the most useful one. It wasn’t a very robust procedure to get to that stage but it built on validated things that other groups had done. I think, as a clinician, there’s always the real, big problem about whether you spend masses of time validating a questionnaire or whether you use other people’s questionnaires but perhaps in a slightly different way.” [020, Trialist/Chief Investigator] |
|  | Developing PROMs to suit research aims and population | “Some of the commercial companies… they’re using third party companies to go out and do qualitative research with patients to identify what are the issues in terms of their quality of life, etcetera, and then turn that into a validated questionnaire which they will then administer as part of their clinical trial. They’re not doing it through PPI, or not what we would normally think of as PPI... I think they think there genuinely is a gap. I don't think they don't want to use existing tools. They actually think there is a gap in that particular therapy area” [041, Trialist/Chief Investigator] |
|  | Role of PPI in selection of relevant PROMs | “It is absolutely necessary to not only have patient reported outcomes but also patient relevant outcomes because if we fail to measure the outcomes that are really meaningful to patients and that really make a difference in their quality of life then, if we fail to do that, we have not sufficient argument when it comes to approving a drug … or maybe later on arguing for reimbursement ... So I think patient involvement is key from the earliest stages of developing those patient reported outcomes.” [039, Journal Editor/Funder/Regulator] |
|  | Effort to ensure PROMs are fit for purpose | “The instruments that we have are not sufficient and it has to do, on one hand, that we now better understand what are the weaknesses of this instrument that we developed years ago, second, we have new treatments, realising that we also have to focus on new, for example, side effects or issues that are relevant to patients, that we have to deal with in these PRO measures. So, yes, we have to make new versions, to make new instruments.” [023, International Expert] |
|  | Developing a clear rationale for using PROM | “The biggest breakthrough, in many ways, for me is concentrating on what is the concept of interest that the intervention is supposed to affect...and then once you’ve identified that, you select the type of measure” [001, International Expert] |
| Barriers | Difficulty selecting a single PROM that matches research aims | “We didn’t feel that we could either choose between some of these measures that were measuring the same thing or not include the whole of the questionnaire. That’s a common challenge, I think, in patient-reported outcome assessment. You either have to back a single questionnaire that doesn’t quite do the job or you put in lots of them and you get some overlapping.” [028, Trialist/Chief Investigator] |
|  | PROM development keeping pace with oncology | “Within the oncology field, the treatments are changing very quickly indeed, so actually patients’ needs and patients’ outcomes are probably changing as well. To spend a lot of time validating this or that questionnaire and then find that, in five or ten years’ time, it’s not relevant anymore is a great shame.” [018, Trialist/Chief Investigator] |
|  | Abundance of PROMs and challenges to data synthesis | “Well depending on the disease you have many… if we talked about neologic dysplastic syndromes, for example, you may use 10 different … questionnaires. So, … I don’t think that having more questionnaires will help because at the end there is also a need to harmonise what we measure” [038, Lived Experience] |
|  | Use of inappropriate PROMs | “Sometimes people are gathering the wrong data… sometimes just put in any old PROMs, they have a whole bunch and they just put them in their studies without really thinking about how they match up to their different objectives.” [008, International Expert] |
|  | Variation in PROM quality | “What worries me, in oncology, is that there are a lot of not very good PROs that are used, especially in terms of speciality cancer spaces and that concerns me.” [011, International Expert] |
